# Author Correction: Dynamics of a qubit while simultaneously monitoring its relaxation and dephasing

**DOI:** 10.1038/s41467-018-05207-3

**Published:** 2018-07-13

**Authors:** Q. Ficheux, S. Jezouin, Z. Leghtas, B. Huard

**Affiliations:** 1 Université Lyon, ENS de Lyon, Université Claude Bernard Lyon 1, CNRS, Laboratoire de Physique, F-69342 Lyon, France; 2 Laboratoire Pierre Aigrain, Département de physique de l’ENS, École normale supérieure, PSL Research University, Université Paris Diderot, Sorbonne Paris Cité, Sorbonne Universités, UPMC Univ. Paris 06, CNRS, 75005 Paris, France; 3 Centre Automatique et Systèmes, Mines ParisTech, PSL Research University, 60 Boulevard Saint-Michel, 75272 Paris Cedex 6, France; 4 QUANTIC team, INRIA de Paris, 2 Rue Simone Iff, 75012 Paris, France

Correction to: *Nature Communications*; 10.1038/s41467-018-04372-9; published online 15 May 2018

The original version of this Article omitted the following from the Acknowledgements: Z. Leghtasʼ primary affiliation is Centre Automatique et Systèmes, Mines ParisTech. This has been corrected in both the PDF and HTML versions of the Article.

