# Supplementary material for: Dynamics of a qubit while simultaneously monitoring its relaxation and dephasing
Source: Nat Commun. 2018 May 15;9:1926. doi: 10.1038/s41467-018-04372-9 (PMC5954145; doi:10.1038/s41467-018-04372-9)
Supplement: Supplementary file 1 — Supplementary Information [file 41467_2018_4372_MOESM1_ESM.pdf]

# Supplementary Information

## "Dynamics of a qubit while simultaneously monitoring its relaxation and dephasing"

Q. Ficheux et al.

## SUPPLEMENTARY NOTE 1 : COMPLETE SET OF EXPERIMENTS

As mentioned in the main text, the experiment was carried out for 30 experimental configurations with $\Omega/2\pi$ ranging from 0 to $(2\ \mu s)^{-1}$ and $\Gamma_d$ ranging from $(30\ \mu s)^{-1}$ to $(300\ ns)^{-1}$. All the experimental results can be visualized in a small animated application available online here (see supplementary figure 1 for an example of the visual)

`http://www.physinfo.fr/publications/Ficheux1710.html`

The measurement can be chosen to take into account the measurement records of the dispersive measurement only, the fluorescence measurement only or both. A direct link to any of the configurations is hidden in each of the panel of Fig. 2, which summarizes the measured distribution of states after 6 $\mu$s. The movies are also available to download directly at

`https://doi.org/10.6084/m9.figshare.6127958.v1`.

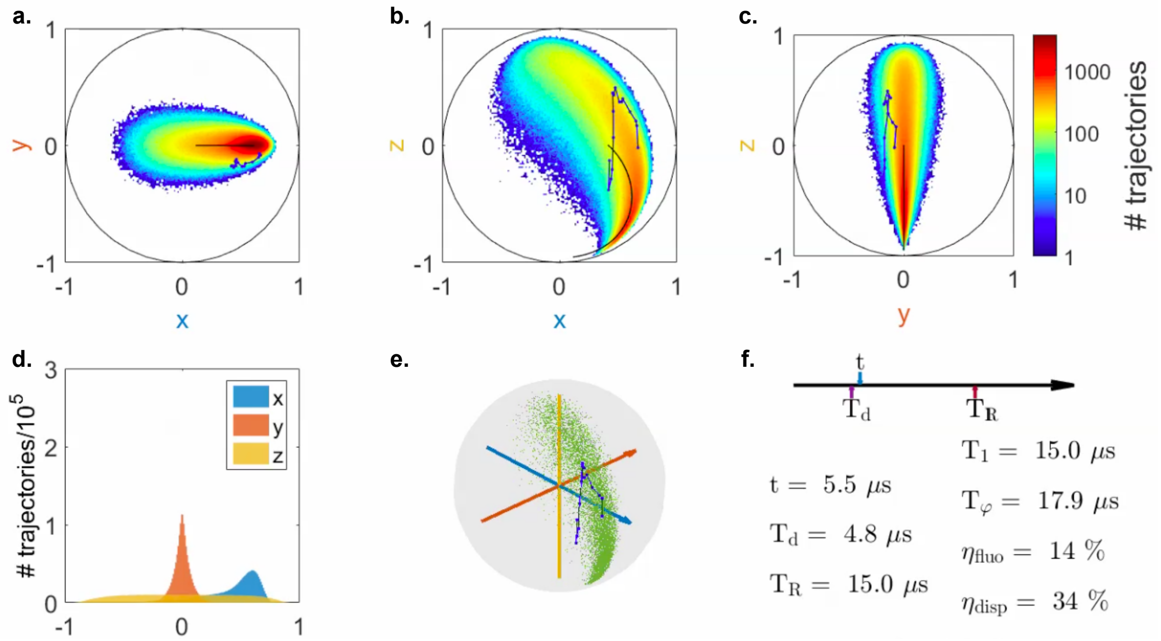


Supplementary Figure 1: Typical visual of the application. Projections of 1.5 million measured trajectories in the $x-y$ (Fig. **a**), $x-z$ (Fig. **b**) and $y-z$ (Fig **c**) planes. The solid black line is the average trajectory, the blue doted line is a randomly chosen trajectory and the color plot is the probability distribution of the trajectories. **d**. Histograms of the $x$, $y$ and $z$ component of the trajectories. **e**. Bloch sphere representation of the probability distribution (green dots) and of the particular trajectory represented in **a**, **b** and **c**. **f**. parameters of the experiment.

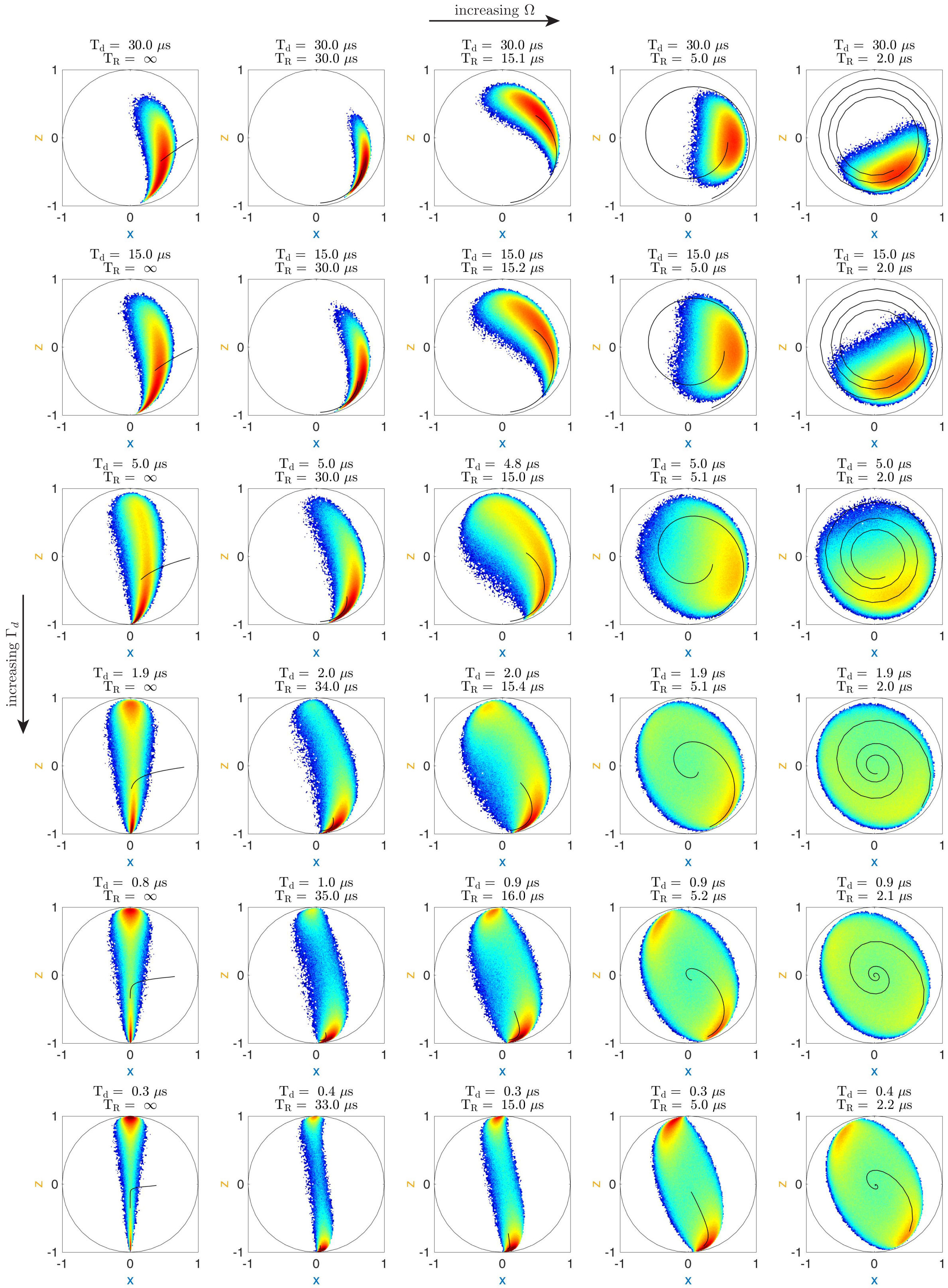


Supplementary Figure 2: Distributions of the $x-z$ Bloch coordinates for 1.5 million measured trajectories shown 6 $\mu$s after the beginning of the measurement sequence. Each panel represents a particular choice of Rabi frequency $T_R^{-1}$ and dispersive measurement rate $T_d^{-1}$. The representation is identical to that of Fig. 1b and both fluorescence and dispersive measurement records are taken into account. **The movie of any of these 30 configurations can be accessed by clicking on the figure.**

## SUPPLEMENTARY NOTE 2 : SYSTEM CHARACTERIZATION AND EXPERIMENTAL SETUP

### Qubit dispersively coupled to a cavity mode

The superconducting qubit was designed according to the standard 3D transmon architecture [1]. It is made of a single aluminum Josephson Junction embedded in a copper cavity of $26.5 \times 26.5 \times 9.5$ mm$^3$ thermalized on the base plate of a dilution fridge at about 20 mK.

In the dispersive coupling approximation, the Hamiltonian of the system reads

$$H = hf_{\mathrm{q}}\frac{\sigma_z}{2} + hf_{\mathrm{c}}a^\dagger a - h\chi\frac{\sigma_z}{2}a^\dagger a \tag{1}$$

with the parameters in Supplementary Table 1, some of which are determined using Fig. 3.

| | |
|---|---|
| $f_{\mathrm{q}}$ (frequency of the qubit) | 5.353305 GHz |
| $f_{\mathrm{c}}$(frequency of the cavity) | 7.7611 GHz |
| $\chi$ (cavity pull) | 5.1 MHz |
| $E_{\mathrm{c}}$ (charging energy) | 174 MHz |
| $\kappa_{\mathrm{out}}$(photon output rate) | $2\pi \times 2.3$ MHz |
| $\kappa_{\mathrm{loss}} + \kappa_{\mathrm{in}}$ (loss rate via non radiative and via input port) | $2\pi \times 320$ kHz |
| $T_1$(lifetime of the qubit) | 15 $\mu$s |
| $T_2$ (coherence time of the qubit) | 11.2 $\mu$s |
| $p_{\mathrm{e}}$ (excitation probability of the qubit) | 2% |

Supplementary Table 1: List of measured qubit and cavity parameters.

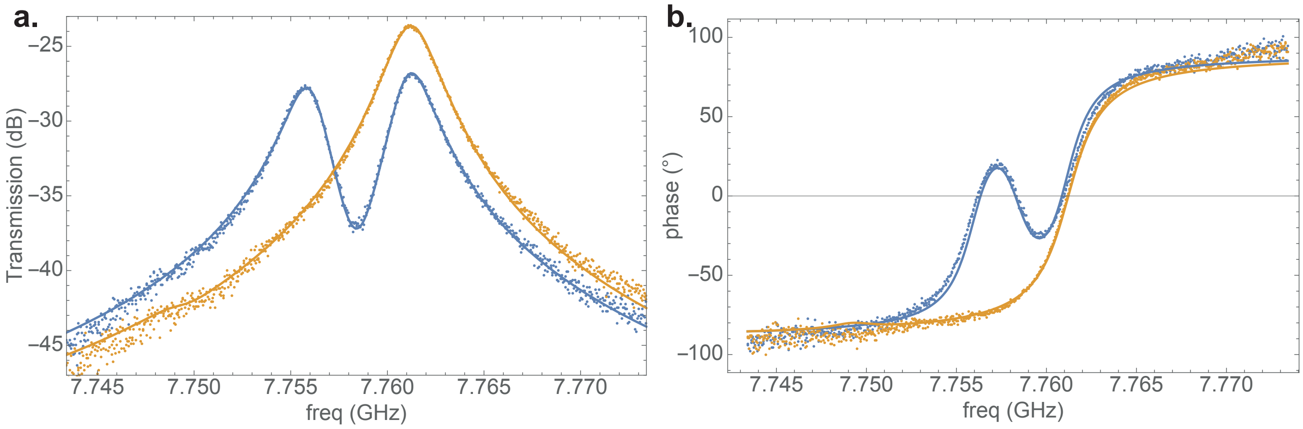


Supplementary Figure 3: Transmission signal through the cavity coupled to the qubit in its ground state (orange dots) and after a $\frac{\pi}{2}$ pulse (blue dots). The amplitude (Fig. **a**) and phase (Fig. **b**) response are fitted with transmission curves across a single mode (solid line) with $\kappa_{\mathrm{out}} + \kappa_{\mathrm{loss}} + \kappa_{\mathrm{in}} = 2\pi \times 2.6$ MHz, $\chi = 5.1$ MHz and $f_{\mathrm{c}} = 7.7611$ GHz. Note that there is an uncalibrated offset on the amplitude that takes into account the attenuation and amplification of the measurement setup.

### Measurement setup

Figure 4 is a diagram of the measurement setup. The qubit-cavity system is probed via two coaxial transmission lines that are each coupled to the 3D cavity through a pin, which extends through the cavity wall. Readout and qubit drives and gates are produced by mixing a continuous wave with 50 MHz (readout) and 40 MHz (qubit) pulses generated by a Tektronix AWG 5014C arbitrary waveform generator. The signal is heavily attenuated and filtered

by cold XMA attenuators and eccosorb-based filters to prevent any thermal noise from reaching the device. At the output of the cavity, a commercial TIGER TGF-A4214-001 frequency diplexer routes output frequencies in the range $DC - 7$ GHz (including the qubit frequency) toward a Josephson Parametric Converter [2] operated as a phase preserving amplifier with gain 17 dB and bandwidth 4.25 MHz. The frequencies 7 GHz $-$ 14 GHz are routed toward a Josephson parametric amplifier (JPA) double pumped by two side-band [3, 4] generated by an IQ mixing of the readout frequency that acts as a phase sensitive amplifier with gain 22 dB and bandwidth 4.25 MHz. The two spatially separated detection chains are further amplified at 4 K (using two Low Noise Factory high electron mobility transistor amplifiers) and also at room temperature before down conversion, digitization by an Alazar 935x ADC board and numerical demodulation. Several cryogenic circulators are used to prevent amplified noise from entering the cavity from the output port and thus increase the qubit temperature and degrade its coherence time.

Both the sample and the amplifiers are shielded from external magnetic fields by aluminium foil and cryoperm boxes and thermally anchored at about 20 mK, the two amplifiers are flux tunable and two superconducting coils (not shown) are located in proximity of the amplifiers to match their operational frequencies with the frequencies of the system.

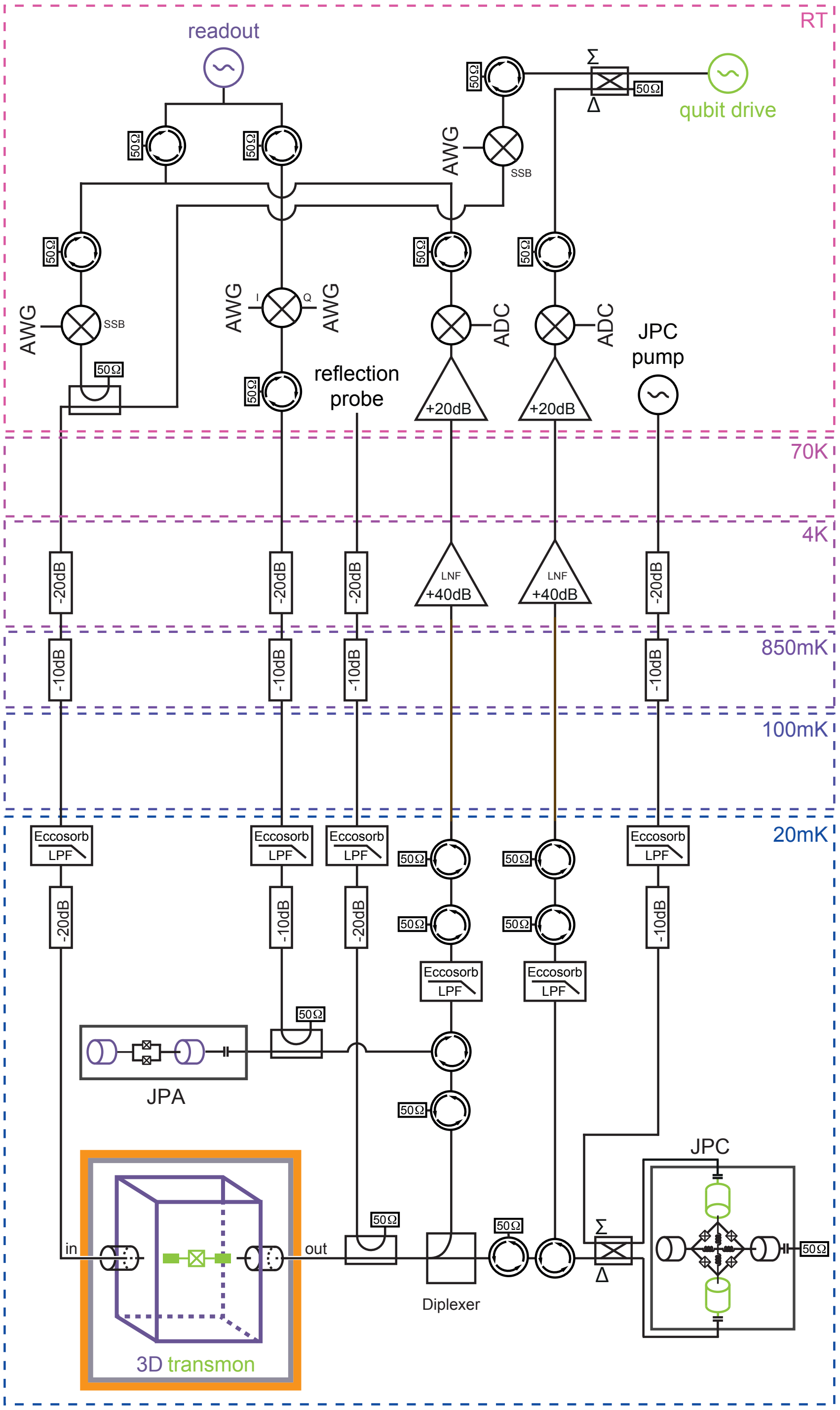


Supplementary Figure 4: Schematics of the experimental setup. A single RF source at $f_c - \chi/2 + 50$ MHz is used to readout the cavity by single side band modulation and to generate a double side-band pump for a Josephson parametric amplifier. The mixed readout signal is sent through the input line, which is heavily attenuated (XMA attenuators) and filtered with home made Eccosorb filters. At the output of the cavity a commercial diplexer routes the signal toward a Josephson parametric converter (JPC) (for frequencies lower than 7 GHz) or a Josephson parametric amplifier (JPA) (for frequencies higher than 7 GHz). The readout pulse is thus amplified by the JPA and sent out of the fridge before down conversion and digitization. An additional tone is mixed at 40 MHz and used for qubit operations and down conversion of the amplified fluorescence field at room temperature before digitization and numerical demodulation. The directional couplers have $-20$ dB coupling.

**Mean Signal**

The raw average of the measurement records gives us the $x$, $y$ and $z$ components of the qubit that are predicted by the Lindblad equation

$$\frac{d\rho_t}{\mathrm{d}t} = i[\frac{\Omega}{2}\sigma_y, \rho_t] + \frac{\Gamma_\mathrm{d}+\Gamma_\varphi}{2}(\sigma_z\rho_t\sigma_z - \rho_t) + \Gamma_\downarrow(\sigma_-\rho_t\sigma_+ - \frac{\sigma_+\sigma_-\rho_t + \rho_t\sigma_+\sigma_-}{2}) + \Gamma_\uparrow(\sigma_+\rho_t\sigma_- - \frac{\sigma_-\sigma_+\rho_t + \rho_t\sigma_-\sigma_+}{2}),$$

where $\Gamma_\uparrow = \frac{1+z_\mathrm{th}}{2}\Gamma_1$ is the excitation rate of the qubit, $z_\mathrm{th}$ is the $z$ component of the qubit at equilibrium and $\Gamma_\downarrow = \frac{1-z_\mathrm{th}}{2}\Gamma_1 \simeq \Gamma_1$ is the desexcitation rate of the qubit.

For an initial state $\rho_0 = \frac{1}{2}(\mathbf{1} + x_0\sigma_x + z_0\sigma_z)$, the time evolution of the component of the Bloch vector reads

$$x(t) = x_\mathrm{inf} + e^{-(3\Gamma_1+2\Gamma_\varphi+2\Gamma_\mathrm{d})\frac{t}{4}}[(x_0 - x_\mathrm{inf})(\cos(\nu t) + \sin(\nu t)\frac{\Gamma_1 - 2\Gamma_\varphi - 2\Gamma_\mathrm{d}}{4\nu} + (z_0 - z_\mathrm{inf})\frac{\sin(\nu t)\Omega}{\nu}] \quad (2)$$

$$z(t) = z_\mathrm{inf} + e^{-(3\Gamma_1+2\Gamma_\varphi+2\Gamma_\mathrm{d})\frac{t}{4}}[(z_0 - z_\mathrm{inf})(\cos(\nu t) - \sin(\nu t)\frac{\Gamma_1 - 2\Gamma_\varphi - 2\Gamma_\mathrm{d}}{4\Omega}) - (x_0 - x_\mathrm{inf})\frac{\sin(\nu t)}{\nu}\Omega], \quad (3)$$

where $\nu = \sqrt{\Omega^2 - \frac{(\Gamma_1-2\Gamma_\varphi-2\Gamma_\mathrm{d})^2}{16}}$, $x_\mathrm{inf} = \frac{2z_\mathrm{th}\Omega\Gamma_1}{\Gamma_1(\Gamma_1+2\Gamma_\varphi+2\Gamma_\mathrm{d})+2\Omega^2}$ and $z_\mathrm{inf} = \frac{z_\mathrm{th}\Gamma_1(\Gamma_1+2\Gamma_\varphi+2\Gamma_\mathrm{d})}{\Gamma_1(\Gamma_1+2\Gamma_\varphi+2\Gamma_\mathrm{d})+2\Omega^2}$.

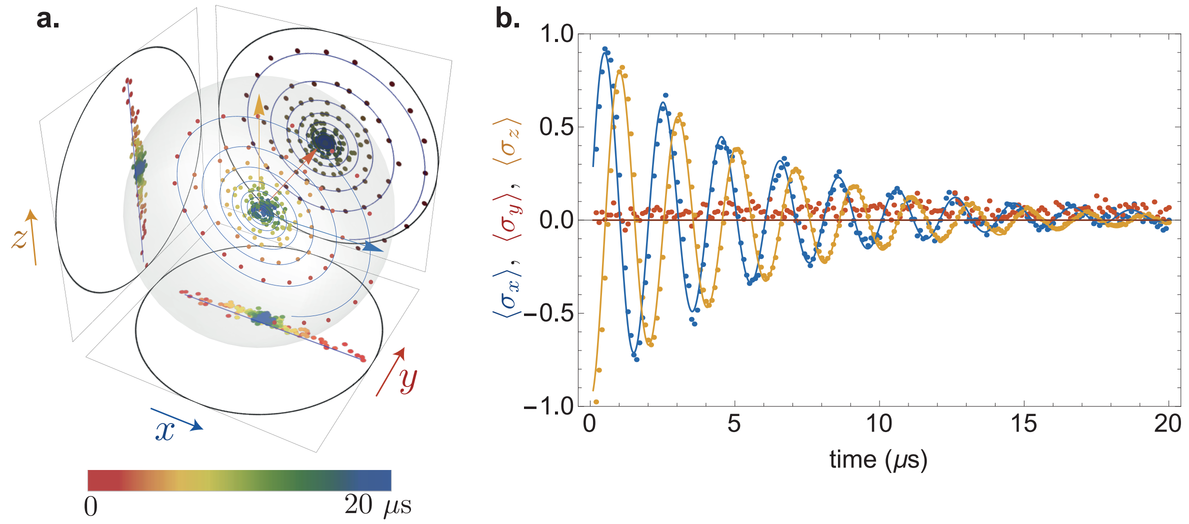


Supplementary Figure 5: Raw average of the measurement records for the experiment corresponding to the dataset of the Fig. **4** in the main text. The solution of the Lindblad equation (solid line) is in excellent agreement with the raw average of the measurement records (dots) for the parameters $\Omega/2\pi = (2\ \mu\mathrm{s})^{-1}$, $\Gamma_\mathrm{d} = (5\ \mu\mathrm{s})^{-1}$, $\Gamma_1 = (15\ \mu\mathrm{s})^{-1}$ and $\Gamma_\varphi = (17.9\ \mu\mathrm{s})^{-1}$. **a.** Bloch sphere representation of the signal. **b.** Projection along the axes of the Bloch sphere.

For every experimental configuration, the values of the parameters $\Omega$, $\Gamma_1$, $\Gamma_\varphi$ and $\Gamma_\mathrm{d}$ were adjusted to make sure that the mean signal and Eqs. (2),(3) match. Indeed the decoherence rates drift over time and depend on the settings of the amplifiers, which also drift over time. At the end of every trajectory a strong measurement is done along $x$, $y$ or $z$ and the result of this tomography was taken into account in the adjustment of the parameters $\Omega$, $\Gamma_1$, $\Gamma_\varphi$ and $\Gamma_\mathrm{d}$ as well.

**Temperature calibration**

In the experiment $z_\mathrm{th} = -0.96$ is the residual $z$ component due to thermal excitation corresponding to a probability of excitation of $p_\mathrm{e} = 1.8\% \pm 0.2\%$ when the qubit is in thermodynamic equilibrium with a bath at $T = 64 \pm 2$ mK. The temperature was estimated by single shot measurement of the $\sigma_z$ component of the qubit at equilibrium (Fig. 6).

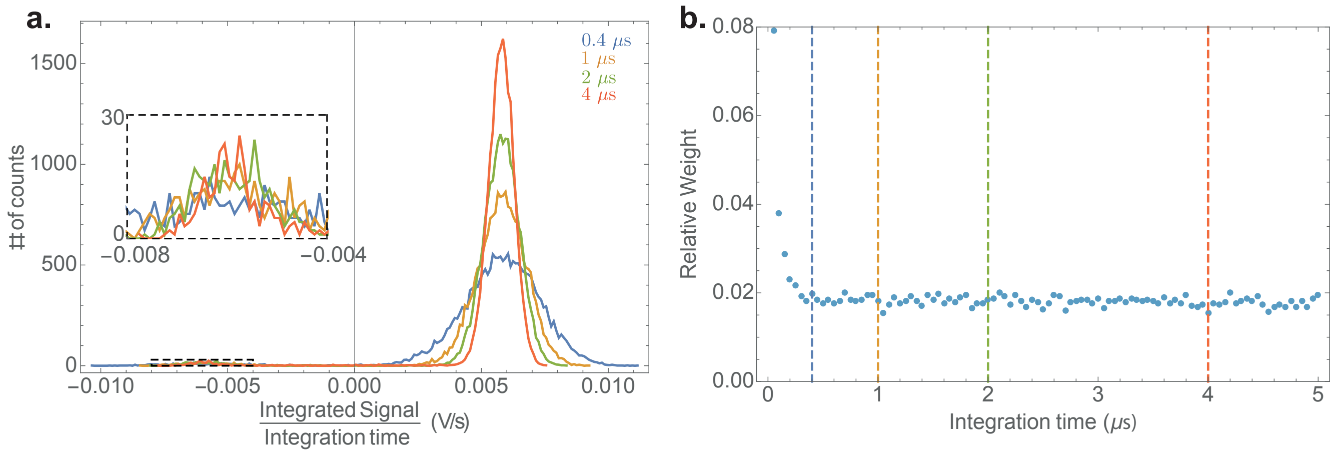


Supplementary Figure 6: Measurement of the temperature of the qubit. **a**. A dispersive readout pulse is applied on the cavity and the in phase component of the signal is integrated for 0.4 $\mu$s (blue curve), 1 $\mu$s (yellow curve), 2 $\mu$s (green curve) and 4 $\mu$s (red curve). Two distinct probability distributions corresponding to the ground state (positive signal) and excited state (negative signal) can be distinguished. **b**. Owing to the finite relaxation time, the relative weight of the two distributions corresponds to the excitation probability only for integration times well below $T_1 = 15$ $\mu$s and well above both the inverse measurement rate $1/\Gamma_{\mathrm{d}} = 0.025$ $\mu$s and the inverse cavity linewidth $\sim 70$ ns.

### Ac stark shift calibration

The dispersive interaction term $-h\frac{\chi}{2}\sigma_z a^\dagger a$ in the Hamiltonian can be understood as a shift of the qubit frequency that depends on the occupation of the cavity. Driving the cavity with a coherent pulse at $f_{\mathrm{c}}$ sets the cavity mode in a coherent state that shifts the frequency of the qubit by an amount $\delta\omega_{\mathrm{Stark}}$ proportional to the number of photons in the cavity [5]. In the experiment, this ac-Stark shift of the frequency of the qubit was precisely measured for each dispersive drive and all trajectories are shown in the qubit rotating frame at the shifted frequency $f_{\mathrm{q}} + \delta\omega_{\mathrm{Stark}}$.

## SUPPLEMENTARY NOTE 3 : COMPARING THE FIDELITY OF A WEAK QUANTUM TOMOGRAPHY AND A PROJECTIVE TOMOGRAPHY

As explained in the paper, a direct averaging of $(u,\ v,\ w)$ on a large number of experiments directly leads to the Bloch coordinate of the density matrix of the system. This tomography protocol highly differs from the usual technique used in Fig. **3** of the paper that consists in measuring the three components of the qubit in separate experiments by projective measurement. It is thus interesting to compare the interest of each technique.

For the case of projective tomography, let us assume that we are able to measure any axis of the bloch sphere with a fidelity $F = 1$. After $3N$ measurements, the probability distribution of the measurement records are given by a binomial distribution of $+1$ and $-1$ and the variance of the estimated Bloch coordinates are $\mathrm{Var}(\langle\sigma_x\rangle_{\mathrm{estimated}}) = \frac{1-x^2}{N}$, $\mathrm{Var}(\langle\sigma_y\rangle_{\mathrm{estimated}}) = \frac{1-y^2}{N}$ and $\mathrm{Var}(\langle\sigma_z\rangle_{\mathrm{estimated}}) = \frac{1-z^2}{N}$. Three million experiments are needed to estimate an arbitrary state with a standard deviation lower than $10^{-3}$.

In the case of tomography based on weak measurements (as in Fig. 2 of the main text), the measurement records are integrated between times $t$ and $t + \mathrm{d}t$

$$\begin{pmatrix} \tilde{u}(t) \\ \tilde{v}(t) \\ \tilde{w}(t) \end{pmatrix} = \begin{pmatrix} \bar{u}(t)/\sqrt{\eta_f \Gamma_1/2} \\ \bar{v}(t)/\sqrt{\eta_f \Gamma_1/2} \\ \bar{w}(t)/\sqrt{2\eta_d \Gamma_{\mathrm{d}}} \end{pmatrix} = \begin{pmatrix} x(t) + \frac{dW_u(t)}{\sqrt{\eta_f \Gamma_1/2dt}} \\ y(t) + \frac{dW_v(t)}{\sqrt{\eta_f \Gamma_1/2dt}} \\ z(t) + \frac{dW_w(t)}{\sqrt{2\eta_d \Gamma_1 dt}} \end{pmatrix}. \quad (4)$$

In the limit of a large number $N$ of experiments $\mathrm{Var}(\langle\sigma_x\rangle_{\mathrm{estimated}}) = \mathrm{Var}(\langle\sigma_y\rangle_{\mathrm{estimated}}) = \frac{2}{N\eta_f\Gamma_1\mathrm{d}t}$ and $\mathrm{Var}(\langle\sigma_z\rangle_{\mathrm{estimated}}) = \frac{1}{2N\eta_d\Gamma_{\mathrm{d}}\mathrm{d}t}$. Thus, with the parameters of Fig. 5, $2 \times 10^9$ experiments are needed to estimate an arbitrary state with a standard deviation lower than $10^{-3}$. This last method is indeed much slower than the standard tomography at least for a small integration time $\mathrm{d}t = 100$ ns as chosen in our experiment.

Nevertheless, the average evolution of a quantum state evolving in time can only be accessed by doing a set of quantum tomography at successive time steps. In Fig. 5, there are 198 time steps of duration $\mathrm{d}t$ so reconstructing the evolution of the state of the qubit by projective tomography would require $6\times10^8$ experiments while $2\times10^9$ experiments are still required for tomography based on weak measurements. The signal to noise ratio of the tomography based on weak measurements is thus independent on the number of successive tomography and it becomes favorable in order to reconstruct lengthy evolution of a qubit.

Another interesting point to note is that the convergence of the result of the projective tomography depends of the state of the qubit. On the other hand the variance of the measurement records in the case of weak tomography is independent of the state of the system.

## SUPPLEMENTARY NOTE 4 : UNRAVELING THE QUANTUM TRAJECTORIES

### From raw data to measurement records

#### *Signal acquired by the ADC*

After the amplification stage, the demodulated signal is integrated over time steps of duration $\mathrm{d}t = 100$ ns leading to correlated and non normalized measurement records $(\tilde{u}_t, \tilde{v}_t, \tilde{w}_t)$ for every discrete time $k\mathrm{d}t$.

#### *Correction of the variations of the gain of the Josephson parametric amplifier (JPA)*

The gain of the JPA on the detection line of the dispersive measurement was fluctuating stochastically over long periods of time with maximum variations of the order of 2.5 dB over the measurement time needed to acquire 1.5 million trajectories. To handle this issue the gain of the JPA was independently measured before and after acquiring every set of 30000 trajectories namely every 5 minutes. The voltage of each measurement record is then rescaled in order to correct for this drift in gain. We also made sure that the quantum efficiencies of the amplifiers are almost independent of the gain over the range of fluctuations.

The source of noise may originate from flux noise induced by trapped vortices on the chip of the amplifier. This hypothesis was consistent with the observation that by warming up the fridge above the superconducting critical temperature $T_c$ of aluminum we observed a substantial reduction of the fluctuation of gain of the amplifier.

#### *Correlations of the output signal*

The JPA and JPC are used as pre-amplifier with a bandwidth of 4.25 MHz $\gg \Gamma_1$ (measured by a Vector Network Analyzer sent on the reflection probe of the cavity) and the signal is integrated over time steps of duration $\mathrm{d}t = 100$ ns. The signals are filtered by the amplifiers and this effect can be modeled as a first order low-pass temporal filter with a time constant $\tau_{\mathrm{JPA}}$ or $\tau_{\mathrm{JPC}}$ and a gain $\beta_{\mathrm{JPA}}$ or $\beta_{\mathrm{JPC}}$ that depends on the gain of the amplifier and that allows us to normalize the variance of the measurement records to $\mathrm{d}t$.

$$
\begin{aligned}
\tilde{u}_t &= \beta_{\mathrm{JPC}}(1 - e^{-\mathrm{d}t/\tau_{\mathrm{JPC}}})u_t + e^{-\mathrm{d}t/\tau_{\mathrm{JPC}}}\tilde{u}_{t-\mathrm{d}t} \\
\tilde{v}_t &= \beta_{\mathrm{JPC}}(1 - e^{-\mathrm{d}t/\tau_{\mathrm{JPC}}})v_t + e^{-\mathrm{d}t/\tau_{\mathrm{JPC}}}\tilde{v}_{t-\mathrm{d}t} \\
\tilde{w}_t &= \beta_{\mathrm{JPA}}(1 - e^{-\mathrm{d}t/\tau_{\mathrm{JPA}}})w_t + e^{-\mathrm{d}t/\tau_{\mathrm{JPA}}}\tilde{w}_{t-\mathrm{d}t}
\end{aligned} \tag{5}
$$

where $(u_t, v_t, w_t)$ would be the normalized records with an infinite bandwidth. The value of the aforementioned parameters were measured, the prefactors $\beta$ are obtained by rescaling the variance of the measurement records to $\mathrm{d}t$ and the correlation times of the filters are chosen to cancel the first order correlations of the measurement records $\mathbb{E}[u_t u_{t-\mathrm{d}t}] = \mathbb{E}[v_t v_{t-\mathrm{d}t}] = \mathbb{E}[w_t w_{t-\mathrm{d}t}] = 0$ (Fig. 7). The time constants are found to be $\tau_{\mathrm{JPA}} = \tau_{\mathrm{JPC}} = 78$ ns and we checked that this condition maximizes the amount of information gathered on the quantum system via the measurement records (highest efficiency that is consistent with tomography results). Note that the equality between the two time constants of the amplifiers occurs owing to a particular choice of power gains for each amplifier. The time step $\mathrm{d}t$ used in the trajectories thus cannot be chosen arbitrary small because of the finite bandwidth of the

amplifier or arbitrary large because the reconstruction with the discrete-time stochastic master equation is only valid for d$t$ much smaller than any time scale involved in the experiment. This last requirement is very important in the Zeno regime, and large deviation between the reconstructed trajectories and independent tomography results were observed for $\frac{1}{\Gamma_\mathrm{d}}$ of the order of d$t$. Thus our choice of d$t$ = 100 ns was the smallest achievable time step compatible with the bandwidth of the amplifiers. The unfiltered and rescaled measurement records were used to reconstruct the trajectories.

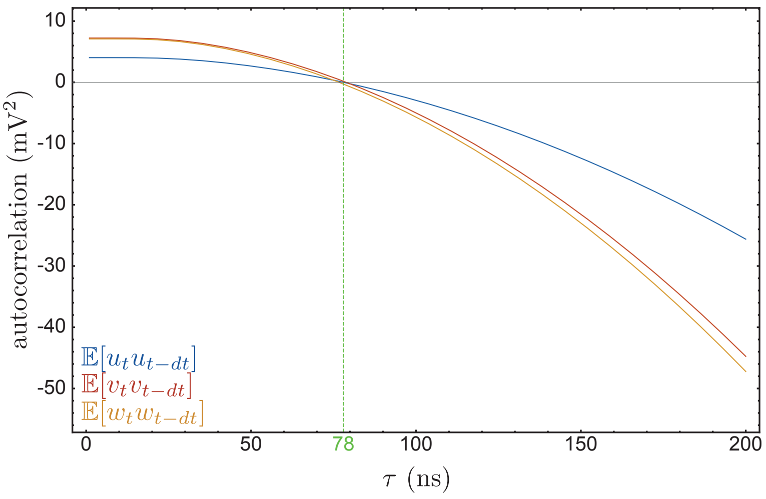


Supplementary Figure 7: Correlations between two consecutive measurement records before normalizing their variance to d$t$ as a function of the time scale $\tau$ of the supplementary Eq. 5 used to cancel the effect of the finite bandwidth of the amplifiers. The parameters $\tau_\mathrm{JPA} = \tau_\mathrm{JPC} = 78$ ns (green dashed line) were found to cancel the autocorrelation of the signals.

## Detection efficiencies

The detection efficiency $\eta_f$ of the fluorescence channels depends both on the probability that the qubit relaxes into the observed output port and on our ability to detect the outgoing electromagnetic signal. The lifetime of the qubit is given by $T_1 = \eta_\mathrm{coll} T_\mathrm{Purcell}$ where $T_\mathrm{Purcell}^{-1}$ is the decay rate associated to spontaneous emission in observed transmission line. The coupling to the output transmission line $\kappa_\mathrm{out}$ was chosen to dominate the rate of internal losses of the cavity and of the unmonitored input line in order to increase the collection efficiency of the setup. The total fluorescence efficiency for the fluorescence measurement is $\eta_f = \eta_\mathrm{coll} \times \eta_\mathrm{JPC}$ where $\eta_\mathrm{JPC}$ is the system efficiency of the heterodyne detection setup and $\eta_\mathrm{coll}$ is the probability that the qubit emits a photon that is collected in the output line during a relaxation event.

Similarly the total efficiency for the dispersive measurement reads $\eta_d = \eta_\mathrm{lines} \times \eta_\mathrm{JPA}$ where $\eta_\mathrm{JPA}$ is the intrinsic detector efficiency and $\eta_\mathrm{lines}$ is an efficiency factor that takes into account losses in the lines and microwave components before the signal reaches the JPA amplifier.

Both $\eta_f$ and $\eta_d$ were first estimated by comparing the mean values of the signals to the amplitude of their fluctuations and these values were confirmed by a self-consistent method (Fig. 8). The trajectories are reconstructed for several values of $\eta_f$ and $\eta_d$ and their coordinates $(x_\mathrm{traj}, y_\mathrm{traj}, z_\mathrm{traj})$ are statistically compared to an independent tomography. The results of the trajectories and the tomography are in agreement only for the correct values of the efficiencies.

We have simulated the experiment in the conditions of Fig. 5 of the main text but with various possible detection efficiencies. This can be done by drawing the stochastic terms d$W_{u,v,w}$ arbitrarily with a normal distribution of variance d$t$ and producing a fictitious measurement record using Eqs. (1,2) of the main text. In Fig. 9b, we see that this procedure accurately reproduces the statistics of trajectories in the case of our experimental efficiencies $\eta_f = 0.14$ and $\eta_d = 0.34$ (Fig. 9a or Fig. 5g,h,i). Then, we show how the expected statistics would spread in the Bloch sphere in case one is able to reach $\eta_{f,d} = 0.5$ (Fig. 9c), $\eta_{f,d} = 0.75$ (Fig. 9d) and $\eta_{f,d} = 1$ (Fig. 9e). As the efficiency increases, the shape of the distribution of states resulting from measurement backaction appear more clearly and the trajectories span a larger area of the Bloch sphere with higher purity states. Interestingly though, the characteristic shapes that are linked to the incompatibility between our various detectors are already qualitatively present at our

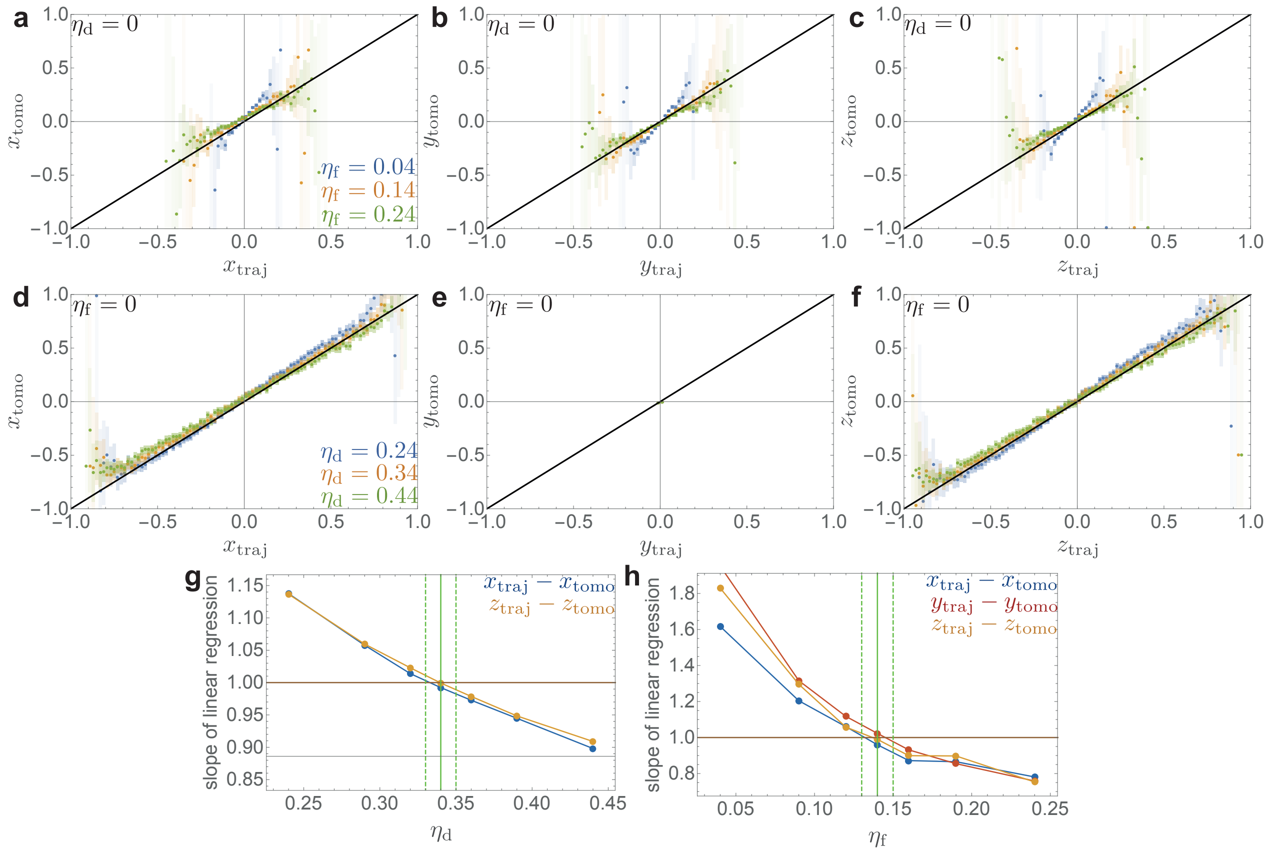


Supplementary Figure 8: Correlations between the coordinates $(x_\mathrm{traj}, y_\mathrm{traj}, z_\mathrm{traj})$ of the trajectories after 19.8 $\mu$s of evolution and an independent tomography on the dataset corresponding to Fig. **4**. In Fig. **a**,**b**,**c** the sole output of the fluorescence records is used to reconstruct the trajectories ($\eta_d = 0$) and $\eta_f$ takes 3 different values. Incorrect values of $\eta_f$ lead to a deviation from a slope one line (in black). Similarly $\eta_f$ is set to 0 in **d**, **e**, **f** and $\eta_d$ is varied. Note that because of the structure of the dispersive measurement ($\sigma_z$ jump operator) back-action, all the dynamics of the qubit is confined in the $x-z$ plane with this sole detection thus $y_\mathrm{traj} = 0$ at all times. In Fig. **g** and **h** the slopes of the correlation histograms are extracted by linear regression for several values of $\eta_f$ and $\eta_d$ used each time in the stochastic master equation. The values $\eta_f = 0.14$ and $\eta_d = 0.34$ (green solid lines) are found by this method with a precision $\pm 0.01$ (green dashed lines).

level of efficiency and only change quantitatively as the efficiencies rise.

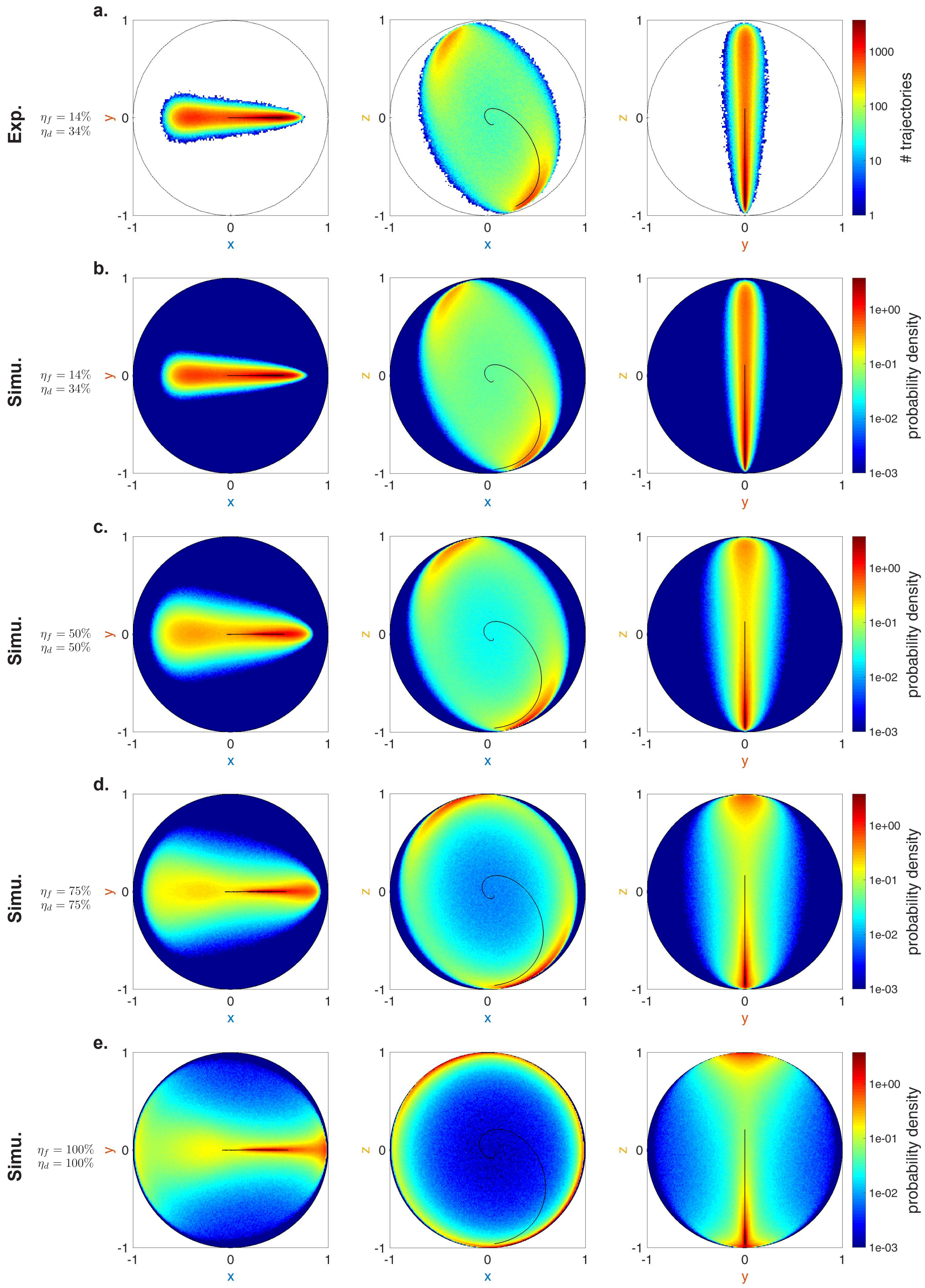


Supplementary Figure 9: Statistics of the projection of qubit trajectories on three planes of the Bloch sphere for measured records or for simulated ones in the configuration of Fig. 5 of the paper. Fig. a reproduces the Figs. 5g,h,i of the main text based on 1.5 millions of actual measurement records. This figure is then reproduced below using simulated measurement records for $\eta_f = 0.14$ and $\eta_d = 0.34$ as in the experiment (Fig. b), $\eta_{f,d} = 0.5$ (Fig. c), $\eta_{f,d} = 0.75$ (Fig. d) and $\eta_{f,d} = 1$ (Fig. e). A clear broadening of the distribution of states occurs for simulated higher detector efficiencies.

### Discrete-time stochastic master equation

The quantum trajectories are reconstructed using a discrete version of the stochastic master equation that is needed to take into account the finite time steps d$t = 100$ ns. It can be written as [6].

$$\rho_{t+\mathrm{d}t} = \frac{\mathbf{K}(\rho_t)}{\mathrm{Tr}(\mathbf{K}(\rho_t)} \tag{6}$$

where the Kraus operator reads

$$\mathbf{K}(\rho_t) = M\rho_t M^\dagger + (1-\eta_d)\frac{\Gamma_\mathrm{d}}{2}\sigma_z\rho_t\sigma_z \mathrm{d}t + (1-\eta_f)\Gamma_\downarrow\sigma_-\rho_t\sigma_+\mathrm{d}t + \Gamma_\uparrow\sigma_+\rho_t\sigma_-\mathrm{d}t + \frac{\Gamma_\phi}{2}\sigma_z\rho_t\sigma_z\mathrm{d}t \tag{7}$$

and

$$M = \mathbf{1} - (iH + \Gamma_\downarrow\frac{\sigma_+\sigma_-}{2} + \Gamma_\uparrow\frac{\sigma_-\sigma_+}{2} + \frac{\Gamma_\mathrm{d}+\Gamma_\phi}{4}\mathbf{1})\mathrm{d}t + \sqrt{\frac{\eta_f\Gamma_\downarrow}{2}}\sigma_-(u(t)+iv(t))\mathrm{d}t + \sqrt{\frac{\eta_d\Gamma_\mathrm{d}}{2}}\sigma_z w(t)\mathrm{d}t \tag{8}$$

A strong numerical advantage for this discrete time method is that the density matrix remains positive, hermitian and of trace one (due to the normalization). The trajectories are then reconstructed step by step from the measurement records.

### Quantum back-action and probability distribution of the trajectories

The stochastic master equation does not clearly show the contribution of the measurement back-action to the dynamics of the system. It is possible to distinguish between two contributions in this equation, the stochastic part is uniquely due to the measurement back-action whereas the deterministic part in d$t$ contains dissipation and the unitary dynamics of the system. During d$t$ the Bloch vector experiences a diffusive step $(\mathrm{d}x_t, \mathrm{d}y_t, \mathrm{d}z_t)$ in the Bloch coordinates that can be decomposed as

$$\begin{aligned}
\mathrm{d}x_t &= \mathrm{d}x_t^\mathrm{stochastic} + \mathrm{d}x_t^\mathrm{dissipation} + \mathrm{d}x_t^\mathrm{unitary}\\
\mathrm{d}y_t &= \mathrm{d}y_t^\mathrm{stochastic} + \mathrm{d}y_t^\mathrm{dissipation} + \mathrm{d}y_t^\mathrm{unitary}\\
\mathrm{d}z_t &= \mathrm{d}z_t^\mathrm{stochastic} + \mathrm{d}z_t^\mathrm{dissipation} + \mathrm{d}z_t^\mathrm{unitary}
\end{aligned} \tag{9}$$

with the stochastic part (Fig. 10)

$$\begin{aligned}
\mathrm{d}x_t^\mathrm{stochastic} &= -\sqrt{2\eta_d\Gamma_\mathrm{d}}x_t z_t \mathrm{d}W_w(t) - \sqrt{\frac{\eta_f\Gamma_1}{2}}\left(x_t y_t \mathrm{d}W_v(t) + (1+z_t-x_t^2)\mathrm{d}W_u(t)\right)\\
\mathrm{d}y_t^\mathrm{stochastic} &= -\sqrt{2\eta_d\Gamma_\mathrm{d}}y_t z_t \mathrm{d}W_w(t) - \sqrt{\frac{\eta_f\Gamma_1}{2}}\left(x_t y_t \mathrm{d}W_u(t) + (1+z_t-y_t^2)\mathrm{d}W_v(t)\right)\\
\mathrm{d}z_t^\mathrm{stochastic} &= -\sqrt{2\eta_d\Gamma_\mathrm{d}}(z_t-1)(z_t+1)\mathrm{d}W_w(t) - \sqrt{\frac{\eta_f\Gamma_1}{2}}(1+z)(x_t\mathrm{d}W_u(t) + y_t\mathrm{d}W_v(t))
\end{aligned} \tag{10}$$

the dissipation part (Fig. 11)

$$\begin{aligned}
\mathrm{d}x_t^\mathrm{dissipation} &= -\left(\frac{\Gamma_1}{2} + \Gamma_\mathrm{d} + \Gamma_\varphi\right)x_t\mathrm{d}t\\
\mathrm{d}y_t^\mathrm{dissipation} &= -\left(\frac{\Gamma_1}{2} + \Gamma_\mathrm{d} + \Gamma_\varphi\right)y_t\mathrm{d}t\\
\mathrm{d}z_t^\mathrm{dissipation} &= -\Gamma_1(1+z)dt
\end{aligned} \tag{11}$$

and the unitary dynamics (Fig. 11)

$$\begin{aligned}
\mathrm{d}x_t^\mathrm{unitary} &= -\Omega z_t\mathrm{d}t\\
\mathrm{d}y_t^\mathrm{unitary} &= 0\\
\mathrm{d}z_t^\mathrm{unitary} &= \Omega x_t\mathrm{d}t
\end{aligned} \tag{12}$$

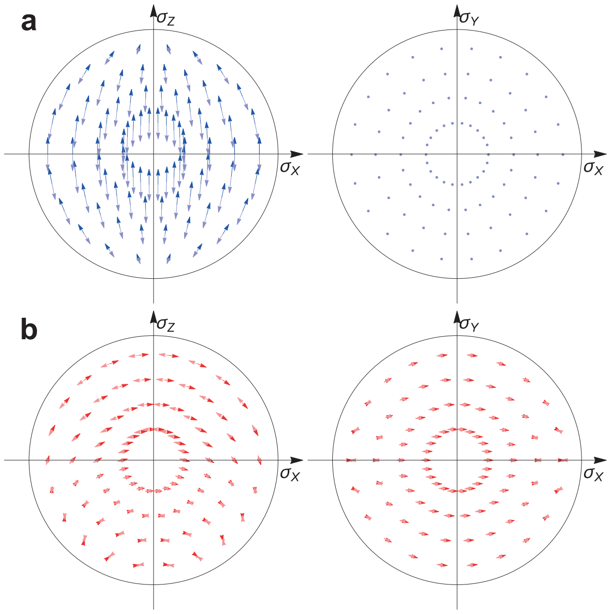


Supplementary Figure 10: Representation of measurement back-action for d$t$ = 100 ns, $\Gamma_1 = (15\ \mu\mathrm{s})^{-1}$, $\Gamma_\varphi = (17.9\ \mu\mathrm{s})^{-1}$, $\Gamma_\mathrm{d} = (15\ \mu\mathrm{s})^{-1}$, $\eta_d = 0.34$ and $\eta_f = 0.14$ in the $\langle\sigma_y\rangle = 0$ plane due uniquely to the stochastic term in d$W_w$ (Fig. **a**) or d$W_u$ (Fig. **b**) in Eq. (10). The middle of the arrow sits at the initial Bloch vector at time $t$ and each double arrow represent two value d$W = +2\sqrt{\mathrm{d}t}$ (dark arrow) and d$W = -2\sqrt{\mathrm{d}t}$ (light arrow). The arrows form a vector field whose field lines end up at the pointer states of the measurement namely $\sigma_z = \pm 1$ for the dispersive measurement (Fig. **a**) and $\sigma_z = -1$ for the fluorescence measurement (Fig. **b**), where no backaction occurs. In the $\langle\sigma_Z\rangle = 0$ plane, the only non zero back-action is associated to the fluorescence measurement. The very different structures of the two back-actions show the incompatibility of the measurements.

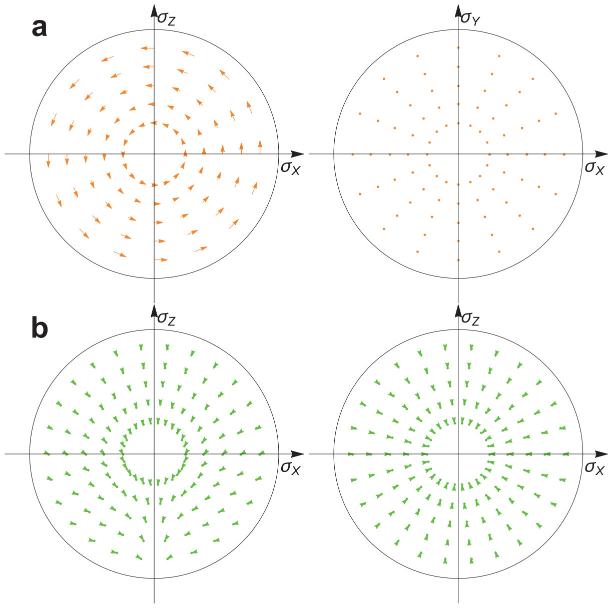


Supplementary Figure 11: Representation of the deterministic evolution that is the sum of the unitary evolution (Fig **a** and Eq. (12)) and decoherence (Fig **b** and Eq. (11)) that are dephasing and energy relaxation for the parameters d$t$ = 100 ns, $\Omega/2\pi = (5\mu s)^{-1}$, $\Gamma_1 = (15\ \mu\mathrm{s})^{-1}$, $\Gamma_\varphi = (17.9\ \mu\mathrm{s})^{-1}$, $\Gamma_\mathrm{d} = (15\ \mu\mathrm{s})^{-1}$.

## SUPPLEMENTARY REFERENCES


[1] P. Campagne-Ibarcq. *Measurement back action and feedback in superconducting circuits.* Ph.D. thesis, Ecole Normale Supérieure (ENS) (2015).

[2] N. Roch, E. Flurin, F. Nguyen, P. Morfin, P. Campagne-Ibarcq, M. H. Devoret and B. Huard. Widely Tunable, Nondegenerate Three-Wave Mixing Microwave Device Operating near the Quantum Limit. *Physical Review Letters* **108**, 147701 (2012).

[3] A. Kamal, A. Marblestone and M. Devoret. Signal-to-pump back action and self-oscillation in double-pump Josephson parametric amplifier. *Physical Review B* **79**, 184301 (2009).

[4] K. W. Murch, S. J. Weber, C. Macklin and I. Siddiqi. Observing single quantum trajectories of a superconducting quantum bit. *Nature* **502**, 211–214 (2013).

[5] J. Gambetta, A. Blais, M. Boissonneault, A. A. Houck, D. I. Schuster and S. M. Girvin. Quantum trajectory approach to circuit QED: Quantum jumps and the Zeno effect. *Physical Review A* **77**, 012112 (2008).

[6] P. Six, P. Campagne-Ibarcq, L. Bretheau, B. Huard and P. Rouchon. Parameter estimation from measurements along quantum trajectories. In *Decision and Control (CDC), 2015 IEEE 54th Annual Conference on*, 7742–7748 (IEEE, 2015).
